# Nicotinamide N-methyltransferase inhibits autophagy induced by oxidative stress through suppressing the AMPK pathway in breast cancer cells

**DOI:** 10.1186/s12935-020-01279-8

**Published:** 2020-05-24

**Authors:** Haitao Yu, Xi Zhou, Yanzhong Wang, Xucheng Huang, Jun Yang, Jin Zeng, Guoli Li, Xinyou Xie, Jun Zhang

**Affiliations:** 1 Department of Clinical Laboratory, Sir Run Run Shaw Hospital, Zhejiang University School of Medicine, 3 East Qingchun Road, Hangzhou, 310016 Zhejiang People’s Republic of China; 2 Key Laboratory of Biotherapy of Zhejiang Province, 3 East Qingchun Road, Hangzhou, 310016 Zhejiang People’s Republic of China; 3 Department of Clinical Laboratory, Xiasha campus, Sir Run Run Shaw Hospital, Zhejiang University School of Medicine, 368 Xiasha Road, Hangzhou, 310018 Zhejiang People’s Republic of China

**Keywords:** Nicotinamide N-methyltransferase, Autophagy, Oxidative stress, AMPK, ULK1, Breast cancer

## Abstract

**Background:**

Nicotinamide N-methyltransferase (NNMT) is highly expressed in several cancers and can regulate cell epigenetic status and various cell metabolism pathways, such as ATP synthesis and cellular stress response. We reported in our previous papers that NNMT overexpression inhibits the apoptosis and enhances the chemotherapy resistance of breast cancer cells. This study aims to investigate the effect of NNMT on autophagy induced by oxidative stress in breast cancer cells, which might provide a novel therapeutic strategy for breast cancer treatment.

**Methods:**

NNMT and LC3B II protein levels in the two cell models (SK-BR-3 and MDA-MB-231) with NNMT overexpression or knockdown were detected by Western blotting and correlated with each other. Changes in cellular viability, intracellular reactive oxygen species (ROS) and ATP levels were assessed after H_2_O_2_ treatment. Then, autophagosomes were imaged by transmission electron microscopy, and LC3 puncta were examined by confocal microscopy and flow cytometry. The LC3B II level and AMPK-ULK1 pathway activity were both detected by Western blotting to determine the role of NNMT in the H_2_O_2_-induced autophagy.

**Results:**

NNMT expression was negatively correlated with LC3B II expression in both cell models (SK-BR-3 and MDA-MB-231). Then, NNMT overexpression attenuated the autophagy induced by H_2_O_2_ in SK-BR-3 cells, whereas knockdown promoted autophagy induced by H_2_O_2_ in MDA-MB-231 cells. Furthermore, mechanistic studies showed that NNMT suppressed the ROS increase, ATP decrease and AMPK-ULK1 pathway activation, resulting in the inhibition of H_2_O_2_-induced autophagy in breast cancer cells.

**Conclusions:**

We conclude that NNMT inhibits the autophagy induced by oxidative stress through the ROS-mediated AMPK-ULK1 pathway in breast cancer cells and may protect breast cancer cells against oxidative stress through autophagy suppression.

## Background

Autophagy is a highly conserved catabolic biological process that enables cells to degrade damaged or unwanted proteins and organelles in lysosomes; thus, it plays a critical role in the recycling of intracellular components and the quality control of proteins and organelles to protect intracellular homeostasis [1, 2]. Although a basal level of autophagy is generally occurs under physiological conditions as part of a cellular repair process, it can be strongly activated in pathological conditions by various stress stimuli, including nutrient starvation and oxidative stress [3], leading to distinct cell fate. Emerging evidence shows that dysfunction of autophagy may lead to a number of diseases, such as metabolic disease and cancer. In cancer progression, autophagy is generally a double-edged sword and its exact role in cancer depends on tumour type, stage, and so on [4]. Recently, much evidence has revealed that the induction or suppression of autophagy can impact cancer status, thus modulating autophagy activity by targeting autophagy regulatory molecules may be a new autophagy-based therapeutic intervention for human cancer treatment [5].

Nicotinamide N-methyltransferase (NNMT), a phase II metabolizing enzyme, mainly transfers a methyl group from S-adenosyl-l-methionine (SAM) to nicotinamide (NAM), producing 1-methylnicotinamide (1MNA) and S-adenosylhomocysteine (SAH). Therefore, NNMT participates in the intracellular methylation cycle, which affects the global methylation status and metabolome of cells [6]. In the past decade, NNMT was found to be highly expressed in many kinds of tumour [7–11] and was found to alter various cancer cell metabolism pathways to regulate the cellular stress response [12, 13] and epigenetic state, which results in high expression of pro-tumour genes [14]. In our previous study, we found that NNMT and its product 1MNA can decrease the mitochondria-mediated apoptosis by suppressing intracellular ROS in breast cancer cells [15]. Recently, we reported that NNMT is overexpressed in breast cancer patients’ tumours and increases the resistance to chemotherapy via its product 1MNA. However, its effect on autophagy regulation in breast cancer has not yet been investigated.

In this study, we examined the expression of NNMT and LC3B II, a marker of autophagy in breast cancer cell line models with NNMT overexpression or knockdown, and then determined correlation between them. Next, we utilized H_2_O_2_ to induce autophagy and detected the levels of autophagosomes, LC3 puncta and LC3B II in cell line models to determine the role of NNMT expression in autophagy regulation. In addition, cell activity, ROS, ATP and autophagy related signalling pathways were also detected to further discover NNMT’s regulation of autophagy induced by H_2_O_2_.

## Methods

### Antibodies

The primary antibodies that included anti-LC3 (#12741), anti-p-AMPK (T172) (# 2535), anti-AMPK (#2532), anti-p-ULK1 (Ser317) (# 12753), anti-ULK1 (# 6439), anti-β-Actin (# 4970) and goat anti-rabbit (# 7074) and goat anti-mouse (# 7076) HRP-conjugated secondary antibodies were all obtained from Cell Signaling Technology (Beverly, Massachusetts, USA). The monoclonal antibody of NNMT was prepared in our lab as previously described [15]. The H_2_O_2_ solution was obtained from Sigma (#H1009).

### Cell lines and cell culture

The human breast cancer cell lines MDA-MB-231, MDA-MB-468, BT549, MCF7 and SK-BR-3 were purchased from American Type Culture Collection (ATCC, USA). Cells were cultured in Dulbecco’s modified Eagle medium (Gibco, USA) containing 10% foetal bovine serum (Gibco, USA) and 100 µg/ml penicillin–streptomycin (Sigma, USA) in a humidified incubator supplemented with 5% CO_2_ at 37 °C.

### Lentiviral vectors and infection

The lentivirus with Plenti-Pur-NNMT or pGCSIL-Pur-shRNA-NNMT vector was purchased from GeneChem Co., Ltd. (Shanghai, China). The lentivirus with the Plenti-Pur-NNMT vector was infected into SK-BR-3 cells to overexpress NNMT, and the lentivirus with the pGCSIL-PUR-shRNA-NNMT vector was infected into MDA-MB-231 cells to silence NNMT. Plenti-Pur and pGCSIL-Pur-shRNA-NC were used as controls. After infection, cells were selected with puromycin (1 µM) for 5 days.

### Western blot analysis

Cell proteins were extracted by lysis buffer (Cell Signaling, USA) with a complete protease inhibitor cocktail (Sigma, USA) on ice. Protein concentrations were quantified using a BCA protein assay kit (Beyotime, China). Eighty micrograms of protein sample was mixed with 5 × loading buffer and loaded into the lanes of 10% sodium dodecyl sulfate–polyacrylamide gel (SDS). The proteins were separated by electrophoresis and transferred to 0.2 μM PVDF membranes (Millipore, USA). After blocking with 5% nonfat milk (Sigma, USA) for 2 h and washing with TBS-T three times at room temperature, the membranes were incubated with primary antibodies at 4 °C overnight. After washing with TBS-T for three times, the membranes were incubated with appropriate HRP-conjugated secondary antibodies for 1 h at room temperature. Protein bands were detected using an ECL detection reagent (Millipore, USA) and imaged by the Image Quant LAS-4000 instrument (Fujifilm, Japan). The band density was analysed by ImageJ and normalized to the level of β-actin in each group. The experiments were repeated independently for three times.

### Cell viability by MTS assay

Cell viability was determined by the MTS assay. The 200 µl cells were seeded into 96-well flat-bottom plates with 8000 cells per well. After incubation for overnight at 37 °C and 5% CO2, cells were treated with H_2_O_2_ (100 or 200 µM) or H_2_O as a control for 8 h. Then, 20 µl MTS reagent (Promega, USA) was added into each well and cells were incubated for another 3 h. Finally, the absorbance value of each well was read at 490 nm using a microplate reader instrument (BIO RAD, Model 680, Japan) and normalized to that of the control group, which was defined as 1. All experiments were performed independently three times using five wells for each group.

### Measurement of cellular reactive oxygen species by flow cytometry

Reactive oxygen species (ROS) were measured by flow assay. Cells were added to 6-well plates and treated with H_2_O_2_ (100 or 200 µM) or H_2_O as a control for 8 h after culture overnight. Then, the cells were washed in HBSS, collected and incubated in 1 mL staining buffer with 10 µl H2DCF-DA (10 mM) (Sigma, USA) for 30 min at 37 °C. Finally, the cells were resuspended in 0.5 mL PBS with 2% FBS after separation from the staining buffer. The fluorescence intensity of H2DCF-DA was analysed by a FACSCalibur flow cytometer (BD, USA) at 485 nm excitation and 530 nm emission to indicate the level of ROS in cells. The data were analysed using Flow Jo 7.5.5 software. The mean fluorescence intensity was normalized to that of the control group, which was defined as 1. The experiment was conducted independently three times.

### Measurement of cellular ATP

The intracellular ATP was measured using the CellTiter-Glo 2.0 reagent kit (Promega, #G9242, USA). Briefly, cells were seeded into the a 96-well clear flat bottom plate (Corning, #3596, USA) with 4000 cells per well. After culturing for 24 h, cells were treated with H_2_O_2_ (100 or 200 µM) or H_2_O as a control for 8 h. Then, 100 µl CellTiter-Glo 2.0 reagent was added into each well. To lyse cells and stabilize the luminescent signal, the plate was mixed on an orbital shaker for 2 min and incubated at room temperature for 10 min. Finally, the value of luminescence in each well was recorded by a luminometer (Turner Designs, TD-20/20, USA) to indicate the ATP level. The mean ATP level was normalized to that of the control group, which was defined as 1. The experiment was performed independently three times.

### Transmission electron microscopy (TEM)

The autophagic vacuoles were examined by transmission electron microscopy. Following H_2_O_2_ (200 µM) treatment or H_2_O as a control for 8 h, cells were fixed with 2.5% phosphate-buffered glutaraldehyde overnight at 4 °C. The next day, cells were post-fixed with 1% osmium tetroxide (OsO_4_) for 1 h after being washed with PBS three times for 15 min. Then, the cells were washed with ddH_2_O and stained with 2% uranyl acetate for 30 min at room temperature. After that, cells were dehydrated with an increasing gradient of ethanol (50%, 70%, and 90%) for 15 min each, ethanol (100%) for 20 min, and 100% acetone for 20 min and embedded into epoxy resin. A 60–80 nm ultrathin section was prepared from each sample by a diamond knife on a Leica Ultracut UCT (Wetzlar, Leica Microsystems GmbH, Germany) and adhered to uncoated 200-mesh copper grids. Then, each sample was stained with 2% uranyl acetate and lead citrate for 15 min each. Finally, each sample was observed by TEM (Tokyo, JEM-1400/JEM-1400 PLUS, Japan) at 80 kV. Autophagic vacuoles were imaged by the transmission electron microscopy (Tokyo, Hitachi H-7000FA, Japan). Images of five view fields in each group were randomly taken for data analysis. The average number of autophagic vacuoles per cell was recorded. The experiment was performed independently three times.

### LC3 puncta formation assay by confocal microscopy and flow cytometry

To analyse autophagic flux, cells were seeded in a 4-well chamber slider (LAB TEK, Rochester, NY) at a density of 2 × 10^4^ cells/well and transfected with a pEGFP-LC3 plasmid (Addgene #24920). Following H_2_O_2_ (200 µM) treatment or H_2_O as a control for 8 h, autophagy was observed under a laser scanning confocal microscope (Olympus). Five representative fields were captured at 200 × magnification, and then the number of LC3 puncta in cells was recorded. The fluorescence intensity of GFP-LC3 in cells after treatment was also analysed by flow cytometry. The mean of fluorescence intensity was normalized to that of the control group, which was defined as 1. The two experiments were both performed independently three times.

### Statistical analysis

All data are presented as the mean ± SEM. The statistical analyses were performed using the SPSS 22.0 statistical software package. Differences between two groups with different treatments were analysed using two-tailed independent-samples Student’s t-test. Statistical significance was defined as *p < 0.05, **p < 0.01, ***p < 0.001 and ****p < 0.0001.

## Results

### NNMT reduces autophagy in breast cancer cells

**Fig. 1:**
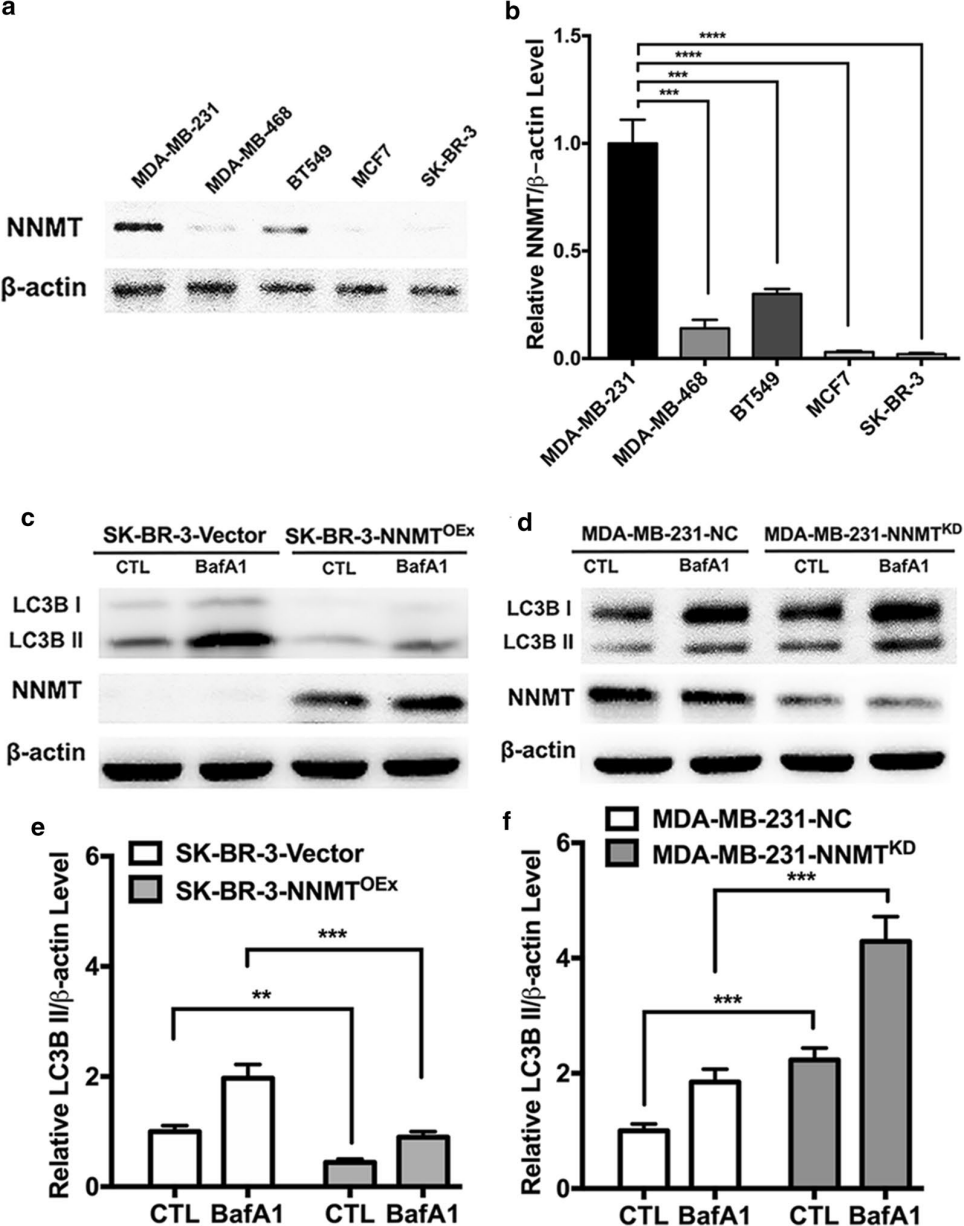
NNMT inhibited autophagy in breast cancer cell lines. **a**, **b** The NNMT protein level was determined in the five breast cancer cell lines by Western blotting. **c**, **d** The LC3B protein level in the two cell models was determined by Western blotting. A representative result from three independent experiments. **e**, **f** The quantification results of (**c**) and (**d**), respectively. The protein levels were normalized to β-actin (**p < 0.01, ***p < 0.001 and ****p < 0.0001)

To determine the effect of NNMT on regulating autophagy in breast cancer cells, we examined the protein level of NNMT in the five cell lines. Among these cell lines, SK-BR-3, MCF7 and MDA-MB-468 showed either no or low NNMT expression, and BT549 showed a medium expression level, while MDA-MB-231 showed the highest level (Fig. 1a, b). Therefore, the SK-BR-3 cell line with no NNMT expression was infected with the lentivirus of Plenti-Pur-NNMT to overexpress NNMT or Plenti-Pur as control, whereas the MDA-MB-231 cell line with the highest NNMT expression was infected with the lentivirus of pGCSIL-Pur-shRNA-NNMT to knockdown NNMT or pGCSIL-Pur-shRNA-NC as control (Fig. 1c, d). These two cell models were successfully used to further study NNMT function in on autophagy regulation. After that, we measured autophagic flux in the two model cell lines by examining LC3B II levels with and without bafilomycin A1 (BafA1), a blocker for autophagy substrate degradation. The LC3B II level was reduced by 50% in SK-BR-3-NNMT^OEx^ cells compared to that in SK-BR-3-Vector cells with or without BafA1 (Fig. 1c, e), which suggests that NNMT overexpression decreased autophagy flux in breast cancer cells. In contrast, NNMT knockdown significantly increased the LC3B II level in the MDA-MB-231 cell model with or without BafA1 (Fig. 1d, f). This result suggested that NNMT reduces autophagy in breast cancer cells.

### NNMT suppresses H_2_O_2_-induced autophagy in breast cancer cells

**Fig. 2:**
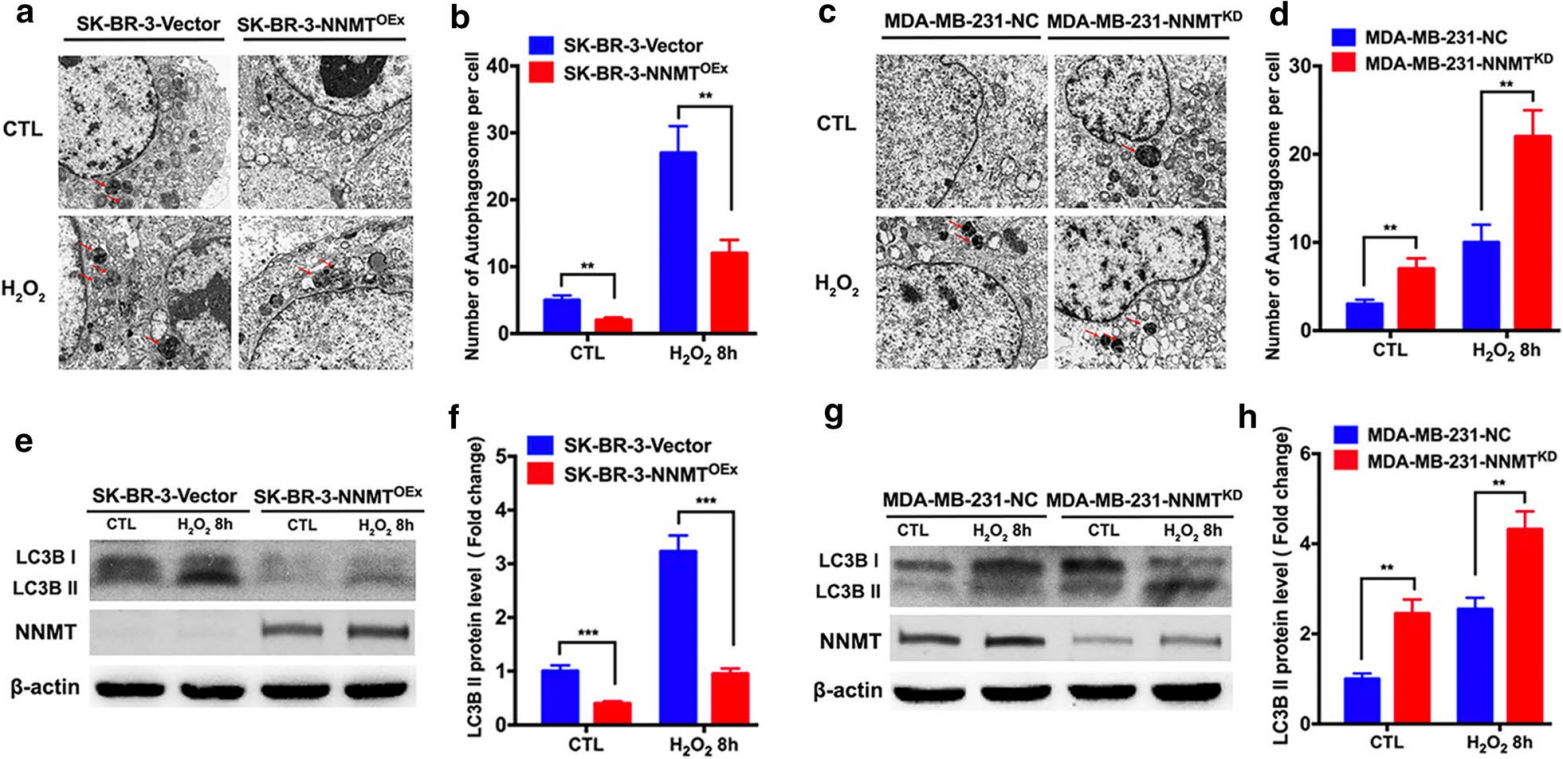
NNMT inhibited autophagy induced by H_2_O_2_. **a**, **c** The autophagosomes in the two cell models after H_2_O_2_ treatment were imaged by transmission electron microscopy. A representative result from three independent experiments. **b**, **d** The quantification results of (**a**) and (**c**), respectively (**p < 0.01 and ***p < 0.001). **e**, **g** The LC3B protein level in the two cell models after H_2_O_2_ treatment was determined by Western blotting. A representative result from three independent experiments. **f**, **h** The quantification results of (**e**) and (**g**), respectively. The protein levels were normalized to β-actin (**p < 0.01 and ***p < 0.001)

To further determine the role of NNMT in autophagy under oxidative stress, we examined autophagy flux in cell models after H_2_O_2_ treatment, which can induce autophagy very well as oxidative stress. The autophagosomes in the two model cell lines after H_2_O_2_ treatment or H_2_O as a control for 8 h were observed by transmission electron microscopy. The number of autophagosomes was lower in SK-BR-3-NNMT^OEx^ cells than in SK-BR-3-Vector cells, whereas a higher number of autophagosomes was observed in MDA-MB-231-NNMT^KD^ cells than in MDA-MB-231-NC cells (Fig. 2a–d), which is consistent with the previous result of the LC3B II assay. With H_2_O_2_ treatment, the numbers of autophagosomes were all increased, indicating that autophagy was induced successfully. The SK-BR-3-NNMT^OEx^ cells showed a significantly lower number of autophagosomes per cell than SK-BR-3-Vector cells, whereas MDA-MB-231-NNMT^KD^ cells showed a significantly higher number of autophagosomes than MDA-MB-231-NC cells (Fig. 2a–d). Consistent with the autophagosome results, the LC3B II level was decreased significantly in SK-BR-3 cells after NNMT overexpression and was increased markedly in MDA-MB-231 cells after NNMT knockdown both with and without H_2_O_2_ treatment (Fig. 2e–h).

**Fig. 3:**
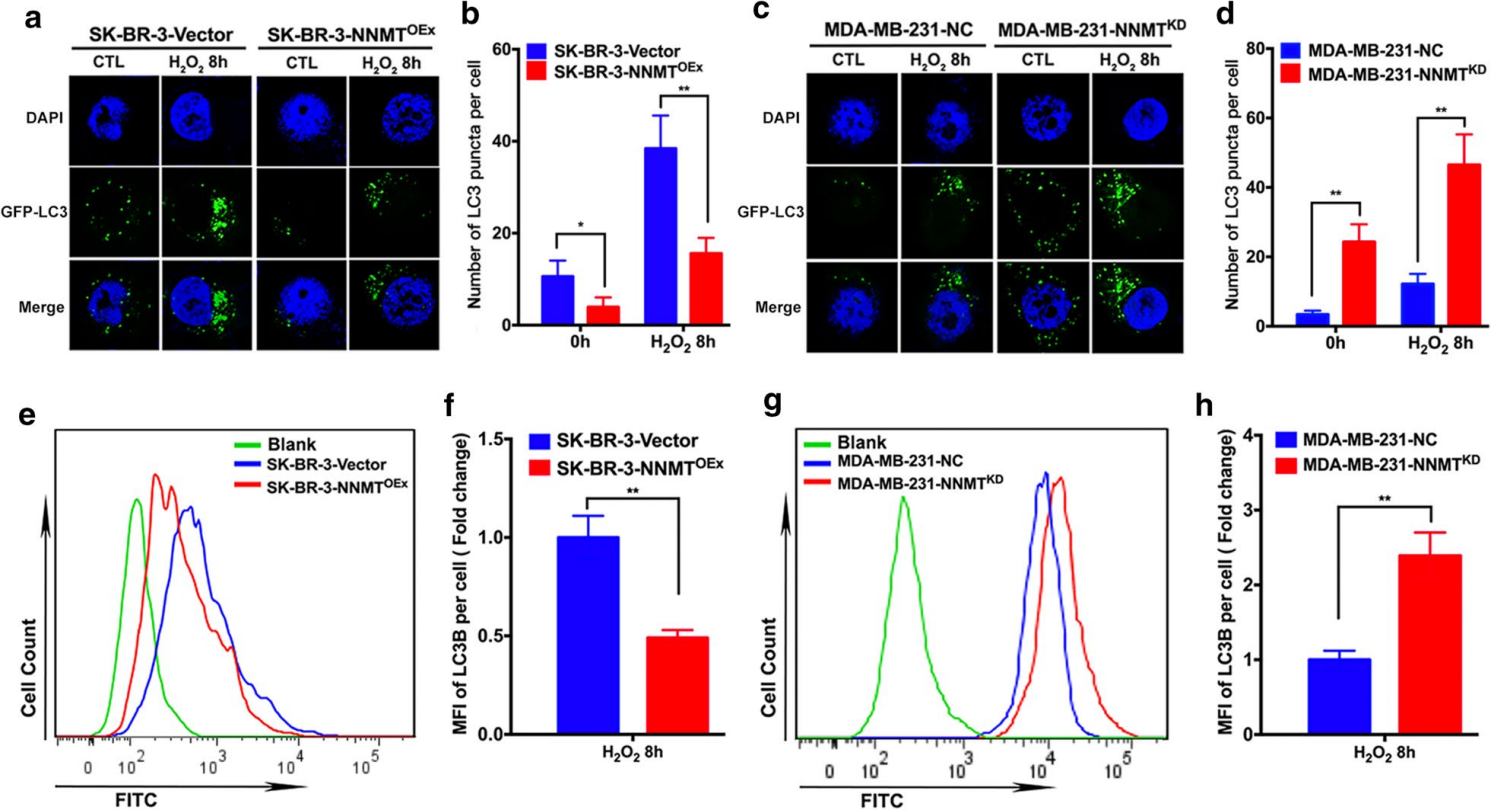
NNMT decreased LC3 puncta formation induced by H_2_O_2_. **a**, **c** The LC3 puncta in the two cell models after H_2_O_2_ treatment were imaged by confocal microscopy. A representative result from three independent experiments. **b**, **d** The quantification results of (**a**) and (**c**), respectively (**p < 0.01 and ***p < 0.001). **e**, **g** The fluorescence intensity of LC3B in the two cell models after H_2_O_2_ treatment was assessed by flow cytometry. A representative result from three independent experiments. **f**, **h** The quantification results of (**e**) and (**g**), respectively (**p < 0.01 and ****p < 0.0001)

Then, we also monitored autophagosomes by confocal and flow assays using the GFP-LC3 plasmid. In the confocal assay, we observed a lower number of LC3 puncta in SK-BR-3-NNMT^OEx^ cells than in SK-BR-3-Vector cells with or without H_2_O_2_ treatment (Fig. 3a, b). In contrast, MDA-MB-231-NNMT^KD^ cells showed the higher number of LC3 puncta than MDA-MB-231-NC cells (Fig. 3c, d). Consistent with the confocal assay results, the mean of GFP-LC3 fluorescence intensity in SK-BR-3-NNMT^OEx^ cells was significantly lower than that in SK-BR-3-Vector cells treated with H_2_O_2_ by flow assay (Fig. 3e, f), whereas MDA-MB-231-NNMT^KD^ cells showed a more than twofold higher mean GFP-LC3 fluorescence intensity than MDA-MB-231-NC cells (Fig. 3g, h). These results indicated that NNMT negatively regulates autophagy induced by oxidative stress in breast cancer cells.

### NNMT decreases the H_2_O_2_-induced autophagy by inhibiting the AMPK-ULK1 pathway in breast cancer cells

**Fig. 4:**
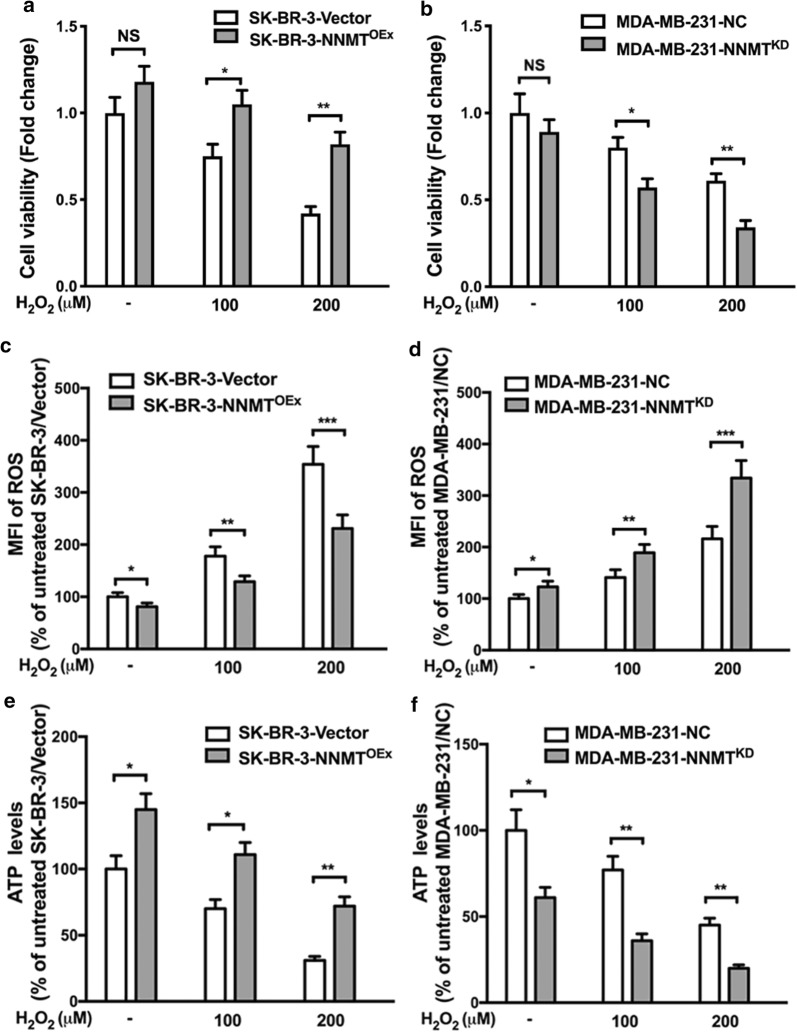
NNMT decreased the inhibition of cell viability and ATP production and the induction of ROS production induced by H_2_O_2_. **a, c, e** The cell viability, intracellular ROS and ATP levels in the SK-BR-3 cell model after H_2_O_2_ treatment were assessed. The data were normalized to the group of SK-BR-3-Vector without H_2_O_2_ treatment, which was defined as 1. **b, d, f** The cell viability, intracellular ROS and ATP level in the MDA-MB-231 cell model after H_2_O_2_ treatment was assessed. The data were normalized to those of the MDA-MB-231-NC group without H_2_O_2_ treatment, which was defined as 1 (*p < 0.05, **p < 0.01 and ***p < 0.001)

**Fig. 5:**
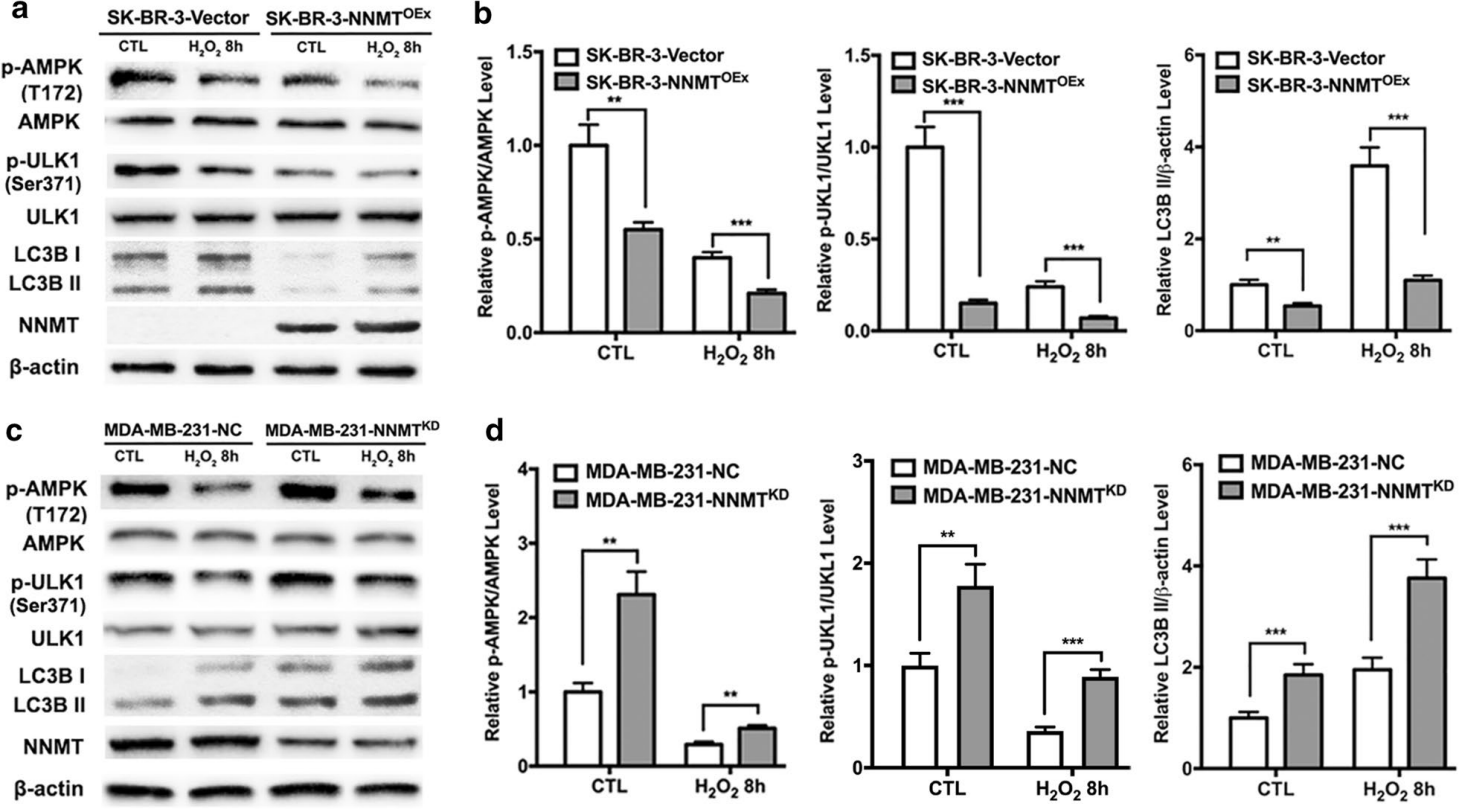
NNMT inhibited H_2_O_2_-induced autophagy by suppressing the AMPK-ULK1 pathway. **a**, **c** The p-AMPK, p-ULK1, AMPK, ULK1 and LC3B protein levels in the two cell models after H_2_O_2_ treatment were determined by Western blotting. A representative result from three independent experiments. **b**, **d** The quantification results of (**a**) and (**c**), respectively. The protein levels were normalized to β-actin (**p < 0.01 and ***p < 0.001)

Given that NNMT is inversely correlated with H_2_O_2_-induced autophagy in breast cancer cells, we further explored the potential mechanisms by which NNMT regulates autophagy. First, we assessed the biological function of NNMT in resistance to oxidative stress. After H_2_O_2_ treatment for 8 h, SK-BR-3-NNMT^OEx^ cells showed the higher cell viability and ATP levels and the lower ROS levels than SK-BR-3-Vector cells. In contrast, MDA-MB-231-NNMT^KD^ cells showed lower cell viability and ATP levels and higher ROS levels than MDA-MB-231-NC cells (Fig. 4). There were all significant differences. These results suggested that NNMT expression decreases the inhibition of cell viability and ATP production and the increases of ROS production, which are related to energy metabolism, resulting in resistance to oxidative stress. Second, we examined the activity of the adenosine monophosphate activated protein kinase (AMPK) pathway after H_2_O_2_ treatment, as it is at the intersection of energy metabolism and autophagy. SK-BR-3-NNMT^OEx^ cells showed a significant lower level of p-AMPK than SK-BR-3-Vector cells both with and without H_2_O_2_ treatment, whereas MDA-MB-231-NNMTKD cells showed a higher level of p-AMPK than MDA-MB-231-NC cells (Fig. 5a–d). Furthermore, SK-BR-3-NNMT^OEx^ cells showed the lower levels of phosphated-serine/threonine protein kinase (p-ULK1) (Ser317), which is phosphorylated by AMPK, than SK-BR-3-Vector cells. Similar to the previous result, the LC3B II level was lower in SK-BR-3-NNMT^OEx^ cells than in SK-BR-3-Vector cells (Fig. 5a, b). In contrast, the p-ULK1 (Ser317) and LC3B II levels were all significantly higher in MDA-MB-231-NNMT^KD^ cells than in MDA-MB-231-NC cells (Fig. 5c, d). These results indicated that NNMT decreases the H_2_O_2_-induced autophagy by inhibiting the AMPK-ULK1 pathway in breast cancer cells.

### NNMT inhibits the activation of the AMPK-ULK1 pathway via its product 1MNA

**Fig. 6:**
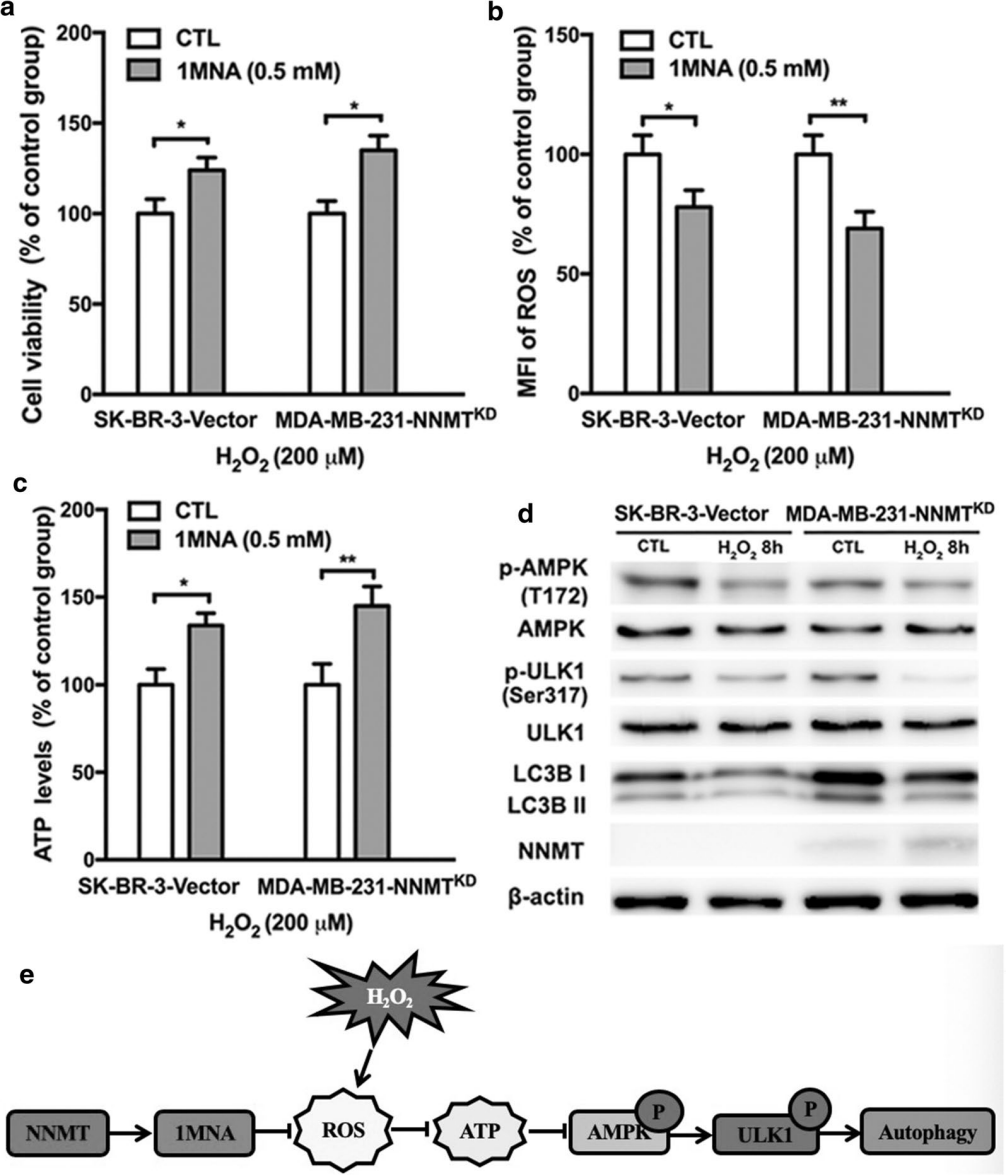
1MNA inhibited autophagy induced by H_2_O_2_ by reducing the inhibition of cell viability and ATP production and the induction of ROS production. **a** The cell viability in SK-BR-3-Vector and MDA-MB-231-NNMT^KD^ cells pretreated with and without 1MNA was assessed by MTS after H_2_O_2_ treatment. **b** The intracellular ROS level in SK-BR-3-Vector and MDA-MB-231-NNMT^KD^ cells pretreated with and without 1MNA was assessed by flow cytometry after H_2_O_2_ treatment. **c** The intracellular ATP level in SK-BR-3-Vector and MDA-MB-231-NNMT^KD^ cells pretreated with and without 1MNA was assessed by an ATP kit after H_2_O_2_ treatment. The SK-BR-3-Vector and MDA-MB-231-NNMT^KD^ groups without 1MNA treatment were considered as the control groups, and their values were normalized to 100%. (*p < 0.05, and **p < 0.01) **d** The p-AMPK, p-ULK1, AMPK, ULK1 and LC3B protein levels in SK-BR-3-Vector and MDA-MB-231-NNMT^KD^ cells pretreated with and without 1MNA were determined by Western blotting after H_2_O_2_ treatment. **e** Schematic model of this study

To further clarify the mechanism, we assessed the change in autophagy level in SK-BR-3-Vector and MDA-MB-231-NNMT^KD^ cells after 1MNA treatment, which is the product of NNMT. The inhibition of cell viability and ATP production and the increase in ROS production induced by H_2_O_2_ were both reduced in SK-BR-3-Vector cells after treatment with 1MNA (0.5 mM). Additionally, cell viability and ATP production were rescued, and the ROS increase was suppressed by 1MNA treatment in MDA-MB-231-NNMT^KD^ cells, similar to NNMT expression (Fig. 6a–c). These results suggested that 1MNA showed resistance to oxidative stress as well as NNMT expression. Then, we examined the activity of the AMPK-ULK1 pathway and LC3B II levels in SK-BR-3-Vector and MDA-MB-231-NNMT^KD^ cells after treatment with 1MNA. In both cell line models, 1MNA treatment reduced the activity of the AMPK-ULK1 pathway and LC3B II levels induced by H_2_O_2_ treatment (Fig. 6d), which indicated that NNMT negatively regulates autophagy under oxidative stress via its product 1MNA.

Together, our results demonstrate that NNMT expression negatively regulates autophagy by inhibiting the activation of the AMPK-ULK1 pathway in breast cancer cells to offer survival advantages to cancer cells under oxidative stress (Fig. 6e). Therefore, NNMT downregulation might be a therapeutic strategy for the combined treatment of breast cancer.

## Discussion

Breast cancer, one of the most prevalent cancers, has the highest mortality rate among cancers in woman worldwide and in China. Therefore, there is an urgent need for new treatment strategies to improve breast cancer treatment.

In recent decades, autophagy has been verified to play a vital role in cancer progression and in response to radiation and chemotherapy, which helps us to shed some light on ways to develop novel strategies for cancer therapy [16]. However, it is generally thought that autophagy has both positive and negative roles in cancer progression, which depends on the particular type of tumour and the stage of cancer progression. In the initial stages, autophagy can inhibit the growth of precancerous cells as a tumour suppressor and decrease the incidence of cancer. During other stages, autophagy may impact the proteome, organelle and metabolism to alter whole cell function. In addition, autophagy also has a survival effect by promoting the adaptation of cancer cells to many stresses, resulting in the resistance to anticancer treatments. Therefore, a comprehensive study of the various contexts involved in the association between autophagy and cancer is beneficial for the novel strategy development of cancer therapy.

Autophagy is a multistep process involving various autophagy-related (ATG) proteins, which are regulated by several upstream signalling pathways, including the AMPK-ULK1 and mTOR pathways [17]. In human cancers, some studies have reported that the ATG genes exhibit low-frequency genomic mutations using large-scale genomic analyses, whereas oncogenic events regulate the cancer-related signalling pathways and consequently lead to abnormal autophagy [18]. One of the earliest discoveries was that p53, a frequently inactivated or mutated transcription factor, can trigger autophagy in cancer cells [19]. The AKT signalling pathway, which promotes cancer cell proliferation, can suppress the TSC1/2 complex, resulting in mTOR1 activation and autophagy inhibition.

NNMT was discovered as a cancer-related protein in recent years. As a methyltransferase, NNMT transfers a methyl group from SAM to NAM to produce 1MNA and SAH, which impacts the global methylation status in cells [20]. NNMT has been recently reported to participate in the development and progression of various carcinomas and regulate cancer metabolism [12]. In breast cancer, we have found that NNMT and its product 1MNA can inhibit the mitochondrion-mediated apoptosis through decreasing intracellular ROS, which enhances the resistance to chemotherapy by SIRT1 protein stabilization [11, 15]. Considering that the NNMT gene was predicted to be an autophagy regulator by genome-wide analysis [21], we investigated the role of NNMT in the autophagy of breast cancer cells.

The regulation of oxidative stress is pivotal in both autophagy and tumorigenesis, since ROS can modulate many signalling pathways involved in autophagy and tumorigenesis through direct or indirect ways [22, 23]. As a second messenger, low to moderate levels of ROS can regulate the activation or expression of multiple signalling proteins such as mitogen-activated protein kinase (MAPK), AMPK, protein kinase B (AKT), extracellular signal-regulated kinase (ERK), c-Jun N-terminal kinase (JNK) [24–29], all of which are involved in cancer cell survival, proliferation and stress response. In a previous study, we reported that NNMT expression increases its product 1MNA level in cancer cells, which decreases intracellular ROS levels to inhibit mitochondria-mediated apoptosis in breast cancer cells and to suppress 5-FU-induced apoptosis in colorectal cancer cells [15, 30]. In this study, even though NNMT inhibited autophagy under normal culture conditions, the NNMT effect was more distinct under oxidative stress. Furthermore, we found that NNMT and its product 1MNA suppress the increase in ROS induced by H_2_O_2_, which may protect mitochondria and cell viability against oxidative stress and inhibit the activation of AMPK induced by oxidative stress in breast cancer cells. AMPK can sense energy changes in cells and maintain cellular energy homeostasis by regulating energy-related metabolism. When the ratio of ATP:ADP in cells is low, AMPK can be activated to directly increase the phosphorylation level of ULK1 at Ser317 and Ser777, which promotes autophagy. Therefore, the AMPK-ULK1 pathway is regarded as the master upstream regulated signalling pathway of autophagy, especially under oxidative and energy stress [31]. In our study, we found that NNMT and its product 1MNA inhibit the activation of the AMPK-ULK1 pathway by suppressing ROS production in breast cancer cells after H_2_O_2_ treatment.

Combined with our results, we hypothesized that NNMT and its product 1MNA negatively regulate autophagy by suppressing the increase in ROS induced by oxidative stress in breast cancer cells. Consistent with our findings, Skin et al. found that NNMT decreases the PP2A methylation level by methyl group transfer from SAM to NAM to inhibit autophagy in liver cancer cells [32]. However, Schmeisser et al. recently reported that NNMT induces autophagy in some animal models by reducing of its substrate SAM [33]. NNMT regulates its substrate SAM and NAM and its product 1MNA levels, which are involved in many important biological processes, including cellular response to stress and cellular energy production. The three molecules maintain a different balance in different types of cells and each of them performs its own function, which may result in the complex function of NNMT in different types of cells. Thus, although NNMT plays a role in regulating autophagy, the detailed mechanisms in different cells require further study.

In addition, cell survival and death are particularly complex processes, and autophagy and apoptosis both play significant roles, between which there is a close association. Our previous and current studies have shown that NNMT plays a role both in regulating apoptosis and autophagy in breast cancer. However, the detailed mechanism by which NNMT regulates the balance between apoptosis and autophagy in breast cancer cells needs further study.

## Conclusions

In this study, we first found that NNMT is involved in autophagy regulation in breast cancer cells. Furthermore, our results suggest that NNMT expression decreases the inhibition of cell viability and ATP production and the increase in ROS production induced by oxidative stress and demonstrated that NNMT and its product 1MNA negatively regulate autophagy to protect breast cancer cells against oxidative stress by suppressing the activation of the AMPK-ULK1 pathway, these findings may provide a novel strategy of targeting NNMT for the combined treatment of breast cancer.

## Data Availability

The datasets generated during the current study are available from the corresponding author on reasonable request.

## Abbreviations

NNMT: Nicotinamide N-methyltransferase
ROS: Reactive oxygen species
SAM: S-adenosyl-l-methionine
NAM: Nicotinamide
1MNA: 1-methylnicotinamide
SAH: S-adenosylhomocysteine
ATCC: American type culture collection
BafA1: Bafilomycin A1
ATG: Autophagy-related
AMPK: Adenosine monophosphate activated protein kinase
MAPK: Mitogen-activated protein kinase
ERK: Extracellular signal-regulated kinase
ULK1: Serine/threonine protein kinase
AKT: Protein kinase B
JNK: c-Jun N-terminal kinase
